# The Asian Nuclear Medicine Board (ANMB); Why Do We Need It?

**Published:** 2013

**Authors:** Durr-e-Sabih

**Affiliations:** MBBS, MSc, FRCP Edin. FANMB. Multan Institute of Nuclear Medicine and Radiotherapy, Multan, Pakistan

Nuclear Medicine faces unique challenges in the 21^st^ century as sophisticated equipment and radiotracers become available to define metabolic processes in ever more exquisite detail. These metabolic processes can be imaged and fused with conventional radiological techniques to provide a synthesis of anatomical and physiological information in the same image set. All of these exciting developments have led to the need of reorienting the very definition of nuclear medicine practice and the required knowledge of the nuclear medicine physician in this century.

In Asia, while some countries are at the forefront of nuclear medicine development, unfortunately there are even more countries which have not kept up with the times nor committed resources to start nuclear medicine (Table 1). There are also questions of “ownership” of the science and art of nuclear medicine and battle lines are being drawn for a turf war in many places. The author has maintained, for over two decades, the urgent need for strengthening nuclear medicine training programs (1). This perception is now shared by many others who believe that the “market acceptance” of the current generation of nuclear medicine physicians depends on broadening the knowledge base by a greater emphasis on complimentary imaging (and therapy) in training programs. In fact, even dual certification is held desirable, if not needed (2). The UK has already started an ambitious program that ensures cross training in radiology and internal medicine for those who opt for the nuclear medicine specialization pathway (3). In addition to all of these, in Asia there are additional issues of standardization in nuclear medicine training, comparable competence and competency/deficiencies (4). Designing a national training program for any country in Asia has challenges that are compounded by varying organizational healthcare structures, needs, material resources and geopolitical stability. Governmental commitment to healthcare varies not only in terms of dollars per person, or percentage of GDP but also in priorities to the extent that in same places nuclear medicine and modern imaging have been relegated to the “nice-to-have” category of facilities rather than “must-have” services (5).

**Table 1:** Nuclear Medicine Practice and Training in Selected ARCCNM* Member States

Country	PAK	MAL	PHI	KOR	SRL	BGD	JPN	THA	IRA	MYA	IND	CPR	VIE	UAE	MON	TWN	INS
certifying examination	Yes	Yes	Yes	Yes	No	Yes	Yes	Yes	Yes	Yes	Yes	Yes	Yes	No	No	Yes	Yes
Nuclear medicine Centres	50	15	30	163	5	20	1267	23	126	5	175	800	20	10	1	49	14
PET imaging Centers	3	6	2	123	1	2	300	8	3	Zero	50	150	5	3	Zero	38	3
Training institutes	15	4	6	24	Zero	1	500	5	3	1	20	80	3	Zero	Zero	35	1
Nuclear Medicine physicians graduating every year	10	4-10	3	24	Zero	5	50	5	6	2	30	200	4	Zero	Zero	6	3
Practicing Nuclear Medicine Physicians	150	30	80	323	5	40	2650	40	320	10	500	6000	40	20	5	171	31
Accrediting Body	Yes	Yes	Yes	Yes	No	Yes	Yes	Yes	Yes	Yes	Yes	Yes	Yes	No	No	Yes	Yes
Numbers are approximate *ARCCNM: Asian Regional Cooperative Council for Nuclear Medicine																	

The Asian Regional Cooperative Council for Nuclear Medicine (ARCCNM), as a body committed to promoting nuclear medicine knowledge in Asia, particularly in developing and less developed countries, has been cognizant of this heterogeneity in the practice and training of nuclear medicine in Asia. The organization has risen to the challenge by establishing an Asian Nuclear Medicine Board (ANMB) that seeks:

- To address growing concerns on the inhomogeneity of training & practice of Nuclear Medicine in Asia.
- To strengthen training programs by developing curriculum of appropriate content that integrates the radiological sciences into molecular imaging.
- To form a resource for use when designing or strengthening national training programs

It was envisaged that board certification will confer upon the nuclear medicine physician an assurance of specialist competence in Nuclear Medicine within the purview of the awarding organization (initially the ARCCNM). A physician who has successfully undergone the process of certification is conferred the title of “Fellow of the Asian Nuclear Medicine Board” (FANMB). Milestones in the evolution of the Board are given in Table 2.

**Table 2:** Milestones in the formation of the Asian Nuclear Medicine Board

i)	At an ARCCNM meeting in Bangladesh (2010) the author proposed an Asian Board in Nuclear Medicine. The proposal was received with great interest.
ii)	Further discussions in Vietnam (2011) and South Korea (2012) led to a commitment by the ARCCNM Executive Council to explore the possibility.
iii)	A core group composed of Drs. Durr-e-Sabih (Pakistan), Henry Bom (South Korea), Jun Hatazawa (Japan) and Theo San Luis (Philippines) was charged with formulating the necessary steps to operationalize this concept.
iv)	A meeting of the core group with representatives of the European Union of Medical Specialists (UEMS) and the European Board of Nuclear Medicine (EBNM) in Vienna (May 2013) concluded with an agreement of support and cooperation between EBNM and ARCCNM.
v)	The core group and executives of the ARCCNM are collecting educational materials and question pool for the examination.
vi)	The first exam is scheduled to be held on November 4, 2014, in Osaka, Japan during the ARCCNM Meeting.

It is understood that the legal standing of the Asian Nuclear Medicine Board (ANMB) will be determined by individual governments. The ANMB on its own will not give the fellow the right to seek employment as a nuclear medicine specialist. The ANMB will provide an objective assessment of skills and knowledge in nuclear medicine but this will not supersede national training certification. Once the caveats inherent

in a regional certification are understood, it becomes easy to appreciate the usefulness of such credentialing. An ANMB certification implies that the requirements in the Region are fulfilled, that weaknesses are being addressed, and that professional development efforts are being implemented. A desire and need for such credentialing is seen by the interest of many Asians and Africans who register for the European Board in Nuclear Medicine (EBNM) outnumbering even those from Europe.

It is noteworthy that the ANMB has sought the cooperation of the European Union of Medical Specialists (UEMS) and European Board of Nuclear Medicine (EBNM) both of which have agreed to send external examiners as well as observers to the first ABNM exam scheduled for November 4, 2014 in Osaka, Japan, preceding the ARCCNM Meeting. This collaboration augurs well for the future of ABNM initiatives.

A core group (Table 2) of Nuclear Medicine physicians from Asia has been mandated with conducting the first ABNM exam and to become the first fellows of the board by first submitting themselves to an examination created by their peers. This will ensure, at the very outset, a commitment to high standards of academic integrity and confidence in the system. Henceforth, the ARCCNM will be responsible for conducting the exam and conferring the fellowship.

The eligibility for registering and taking the Board examination has purposefully been kept easy. Any medical graduate, registered with the national medical council or association, who has 5 years or more experience in clinical nuclear medicine would be eligible to sit for the exam. This experience might be while still on a training program or already in practice. Moreover, ARCCNM shall provide some financial assistance to candidates accepted to sit for the exam. Furthermore, registration fees for the ARCCNM Meeting will also be waived off for selected candidates. Finally, ARCCNM will post the curriculum contents and educational material on its website to include basic nuclear medicine material and topics of special interest or importance for the board exam e.g., cross sectional imaging, therapy, new directions, etc.

The Asian Nuclear Medicine Board is expected to go a long way in providing educational resources and certifications in Nuclear Medicine that are appropriate for Asian specialists. It is expected that this certification will be a source of pride for its fellows as it enhances confidence among employers, peers and patients alike that the fellow has demonstrated his competency in an exam that is at par with the best in the world.
